# The role of recombinant epidermal growth factor and serotonin in the stimulation of tumor growth in a SCCHN xenograft model

**DOI:** 10.3892/or.2012.1903

**Published:** 2012-07-06

**Authors:** CHRISTIN GEISSLER, MARKUS HAMBEK, ANNE ECKARDT, CHRISTOPH ARNOLDNER, MARC DIENSTHUBER, TIMO STÖVER, JENS WAGENBLAST

**Affiliations:** 1 ENT Department, Medical School, Goethe University, Frankfurt am Main, Germany; 2 ENT Department, Medical University of Vienna, Austria

**Keywords:** squamous cell carcinoma of head and neck, epidermal growth factor, serotonin, tumor growth in xenografts

## Abstract

One challenge of squamous cell carcinoma of the head and neck (SCCHN) chemotherapy is a small percentage of tumor cells that arrest in the G0 phase of the cell cycle and are thus not affected by chemotherapy. This could be one reason for tumor recurrence at a later date. The recruitment of these G0-arresting cells into the active cell cycle and thus, proliferation, may increase the efficacy of chemotherapeutic agents. The aim of this study was to investigate whether stimulation with recombinant epidermal growth factor (EGF) or serotonin leads to an increased tumor cell proliferation in xenografts. Detroit 562 cells were injected into NMRI-Foxn1nu mice. Treatment was performed with 15 μg murine or human EGF, or 200 μg serotonin. The control mice were treated with Lactated Ringer’s solution (5 mice/group). Tumor size was measured on days 4, 8 and 12 after tumor cell injection. The EGF stimulated mice showed a significantly higher tumor growth compared to the serotonin-stimulated mice and the untreated controls. In the present study, we show that it is possible to stimulate tumor cells in xenografts by EGF and thus, enhance cell proliferation, resulting in a higher tumor growth compared to the untreated control group. In our future investigations, we plan to include a higher number of mice, an adjustment of the EGF dosage and cell subanalysis, considering the heterogeneity of SCCHN tumors.

## Introduction

Squamous cell carcinoma of the head and neck (SCCHN) is the 6th most common cancer type worldwide (1). The majority of cancers of the upper aerodigestive tract are of squamous cell origin (2). In Germany, >15,000 new cases of oral, pharyngeal and laryngeal carcinoma are reported each year (3). It is well-known that tobacco and alcohol consumption, as well as human papillomavirus (HPV) infection are associated with the development SCCHN.

The epidermal growth factor receptor (EGFR) is a basic cellular regulator of essential functions that regulate the survival, migration and proliferation of cells. EGFR signaling is impaired in various cancers. In SCCHN, EGFR is found to be overexpressed in 90% of cases (4). Its overexpression is an early event in SCCHN tumorgenesis and correlates with poor prognosis (4,5). Over the past decade, the knowledge of EGF overexpression in these tumors has led to the introduction of antibodies targeting EGFR for the treatment of head and neck cancer. For example, drugs such as the EGFR antibody, Cetuximab^©^ or the tyrosine kinase inhibitors, Gefitinib^©^ and Erlotinib^©^, target EGFR and are often used in SCCHN therapy (6). The EGF ligand stimulates the proliferation of keratinocytes. Therefore, EGF promotes DNA synthesis and the progression from the G1 to the S phase of the cell cycle (7). Another mediator of the cell cycle is serotonin (5-HT). 5-HT is a growth factor, which regulates DNA synthesis. The mitogenic effect of 5-HT is thought to act via 5-HT1A and 5-HT1B (8).

In SCCHN therapy, platinum agents (cisplatin and carboplatin), taxanes (docetaxel) and antimetabolic agents (5-fluorouracil) are commonly used as chemotherapeutic drugs. These cytostatic drugs affect cells in the S or M phase. One challenge of SCCHN chemotherapy is that a small percentage of tumor cells do not proliferate (9). These cells arrest in the G0 phase of the cell cycle and are not affected by chemotherapy. This could be one reason for tumor recurrence at a later date. The recruitment of these G0-arresting cells into the active cell cycle and thus, proliferation, may increase the efficacy of chemotherapeutic agents.

Former *in vitro* experiments performed by Hambek *et al*(10), have provided evidence that the treatment of tumor cells with EGF and 5-HT can decrease the amount of dormant G0/G1 cells, resulting in more active, dividing cells that are consequently more sensitive to chemotherapeutic treatment. The aim of this study was to investigate whether tumor cell stimulation with EGF and 5-HT can affect tumor growth in xenografts.

## Materials and methods

### Cell culture

Detroit 562 cells (CCL-138; American Type Culture Collection) were cultured in Eagle’s minimum essential medium (10% FCS, 0.5 mM sodium pyrovate, 25 mg gentamycin) at 37°C and 5% CO_2_. For injection, cells were detached with Accutase (PAA Laboratories) and the concentration of living cells was determined using Cedex XS cell counter (Innovatis). The cells were diluted in Lactated Ringer’s solution in a concentration of 5×10^6^ cells/0.1 ml. The injection solution was transferred on ice to where the animals were housed.

### Mice, tumor xenografts and treatment

Mice were housed in a pathogen-free facility for a 12-h light-dark cycle and with free access to food and water. Six-week-old female NMRI-Foxn1nu mice (Harlan) were anesthetized with forane (Baxter) evaporated with Dräger Forena Vapor (19.3). Five million cells (100 μl) were subcutaneously (s.c.) injected into the flank of each mouse. One day after tumor cell injection, treatment was performed with 15 μg EGF (murine EGF; mEGF) (315-09; PeproTech), human EGF (hEGF) (100-009; RELIATech GmbH), or 200 μg serotonin (B21263; Alfa Aesar). The control mice were treated with Lactated Ringer’s solution. Each treatment group consisted of 5 mice. Mice were treated as described above, daily for a period of 10 days. The tumor size was measured on days 4, 8 and 12 after tumor cell injection using a digital caliper. The tumor volume was calculated with the following formula: V = π/6 × length × width^2^. All mice were sacrificed by the 12th day after tumor cell transplantation or before the tumors ulcerated.

### Staining

After sacrifice, tumors were etched. One tumor was directly frozen in liquid nitrogen and the second was fixed in Notoxhisto (Quartett) and embedded in paraffin. Ki67 and EGFR staining was performed on the frozen sections. Immunohistological staining for CD31 was carried out on the paraffin-embedded sections. CD31 is a marker for lymphatic and blood vessels. Ki67 (rabbit, dilution 1/200) (Ki681C01; DCS), EGFR (rat, dilution 1/200) (ab231; Abcam) and CD31 (rat, dilution 1/20) (DIA-310; Dianova) primary antibodies were used for the staining procedure. Incubation was carried out for 1 h at room temperature. Afterwards, we proceeded with the DCS Detection Line system (AD050POL-K, PD000POL-R), according to the supplier’s instruction. Staining was performed with DAB reagent (DC137C100). The Fuchsin Substrate-Chromogen system (K0625; Dako) and HistoGreen (E109; Linaris). Images were taken under a Zeiss Axioplan 2 with an AxioCam ICc1 camera. Statistical analysis was performed with BIAS for windows version 9.12 using one-way ANOVA. The animal experiments were approved by Regierungspräsidium Darmstadt, Hessen F66/08.

## Results

### Increased volume in EGF-treated tumors

**Figure 1:**
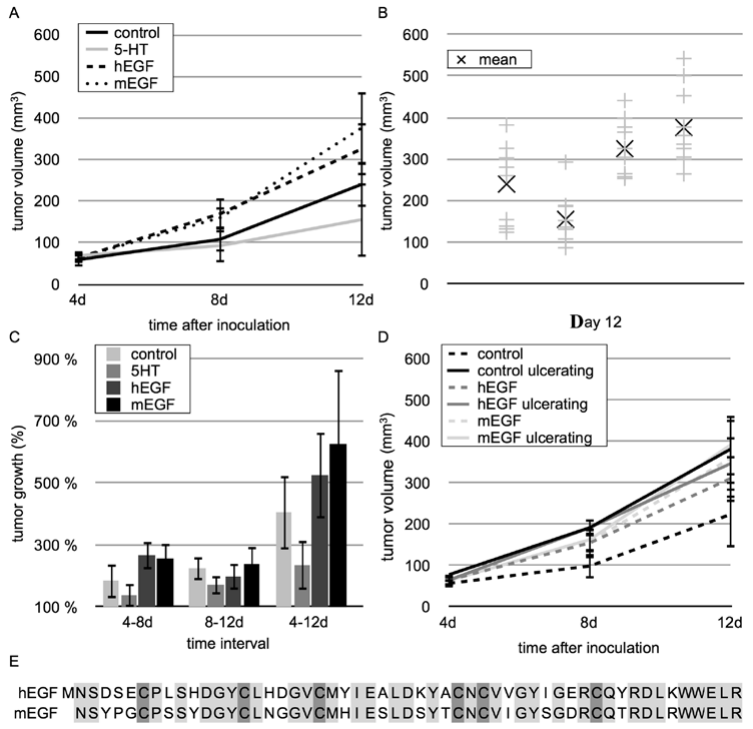
Growth of tumor xenografts in hEGF-, mEGF-, 5-HT-treated mice and controls. (A) Tumor enlargement; (B) size of tumors on day 12; (C) growth increase of tumors in 4-day time intervals; (D) enlargement of ulcerating and non-ulcerating tumors; (E) amino acid sequence of hEGF and mEGF.

The daily injection regime of EGF led to an enhanced tumor volume in both groups of mEGF- and hEGF-injected mice (Fig. 1A). After 12 days, the mean tumor volume in the hEGF-treated mice reached 325±63 mm^3^, whereas in the control mice, the mean tumor volume was only 240±89 mm^3^. The mean tumor volume in the mEGF-treated mice was 376±88 mm^3^, which was comparable to that of the hEGF-treated mice (Fig. 1B).

In all tumors, the rate of intratumoral connective tissue was constant at 33–67% of the tumor tissue (±4%, data not shown) indicating that growth increase was caused by tumor proliferation and not by edema or an increase in connective tissue. The maximal growth increase was measured between days 4 and 8 (Fig. 1C). During this time, hEGF-stimulated tumors grew by 267±46%, the tumors of the mEGF-treated mice grew by 257±47% and the control tumors only increased by 185±38%; however, this did not reach statistical significance (p>0.05). The tumors of the 5-HT-treated mice showed a lower growth increase compared to the control tumors and reached a final volume of 155±54 mm^3^ (Fig. 1A).

### EGF treatment increases the risk of tumor ulceration

**Figure 2:**
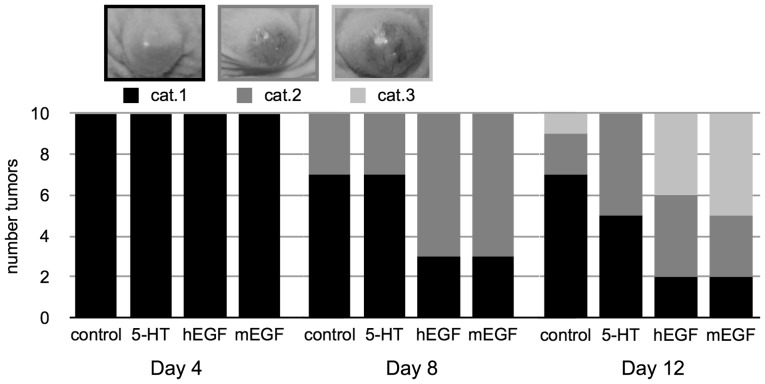
Ulceration of mouse tumors; cat. 1, unremarkable or slight shading; cat. 2, marbled stain and spots under the skin; cat. 3, ulcer. For details see Materials and methods.

The experiment was terminated by the 12th day or when tumor ulceration occured. After this treatment period, 1 control tumor, 4 tumors from the hEGF-treated mice and 5 mEGF-treated tumors had ulcerated (Fig. 2). This destruction of the surface epithelium is a common event in SCCHN patients (11) and also in xenograft models. We graded the ulceration process into 3 categories. At the beginning, the tissue beyond the tumor was unremarkable or showed a rose shading and was assigned to category 1 (cat. 1). Category 2 (cat. 2) was characterized by red marbling and violet spots under the skin. Finally, tumor ulceration occured [category 3 (cat. 3)]. In our experiment, the tumors of the EGF-treated mice ulcerated more frequently than those of the control group.

**Figure 3:**
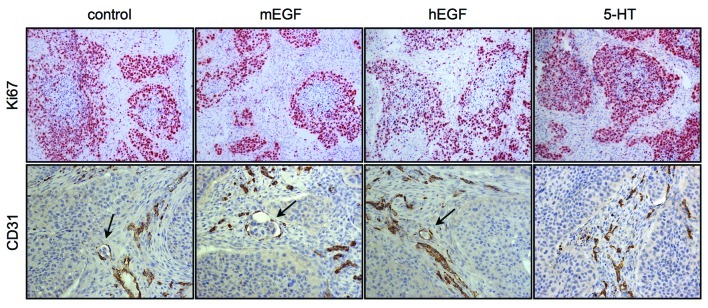
Immunohistological staining of control, hEGF-, mEGF- or 5-HT-treated mice for Ki67 and CD31. Arrows indicate vessel invasion.

The amount of vessels in the intratumoral connective tissue remained equal in the control and hEGF-treated tumors (6±4%, data not shown) (sample images shown in Fig. 3). In the 5-HT-treated mice, the tumor ulceration rate was comparable to that of the control mice. We did not observe any vessel invasion in this group (Fig. 1D).

### The majority of xenograft tumor cells express EGFR and Ki67

**Figure 4:**
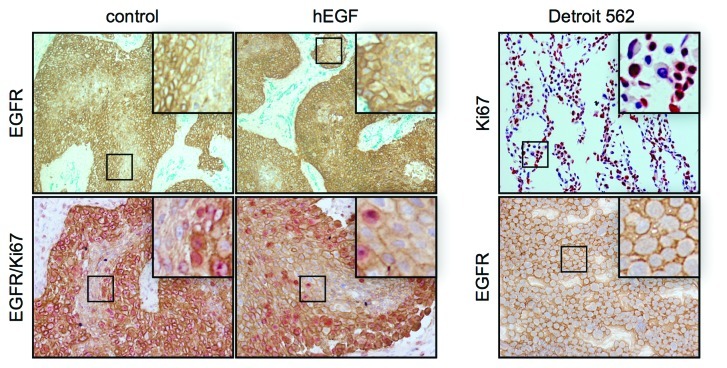
Immunohistological staining of control and hEGF-treated mouse tumors, EGFR/CD31 and EGFR/Ki67 double staining, staining of Detroit 562-cultured cells for Ki67 or EGFR. Quadratic area is enlarged, EGFR (brown), CD31 (green), Ki67 (red).

Detroit 562 cells overexpress EGFR (12). Immunohistological staining of EGFR indicated that high levels of EGFR were present in the cell cultures and tumor xenografts (Fig. 4). Almost all tumor cells expressed EGFR. *In vitro*-cultured Detroit 562 cells had an equal amount of EGFR in each cell. In the mouse xenografts, the level of EGFR expression varied in the cells. The EGFR expression seemed to depend on the localization of the cell. Tumor cells that were located next to the necrotic core of the tumor cell nests had lower quantities of EGFR. By contrast, most cells at the borders had a high EGFR expression. The reduction in EGFR was potentially caused by necrosis or tumor cell differentiation. In the skin, for instance, the amount of EGFR is reduced during the differentiation process (7).

Seventy-four percent of the *in vitro*-cultured Detroit 562 cells were Ki67-positive (Fig. 4). In the tumor xenografts, the majority of tumor cells was also Ki67-positive. The results showed that these cells were in the active cell cycle. However, a number of Ki67-negative cells was localized in the middle of the tumor cell nests. These cells were supposed to be quiescent in the G0 phase. The size of cell nests correlated with the number of inner Ki-67-negative cells. In the EGF-stimulated mice, the number of Ki-67-positive cells was much higher.

**Figure 5:**
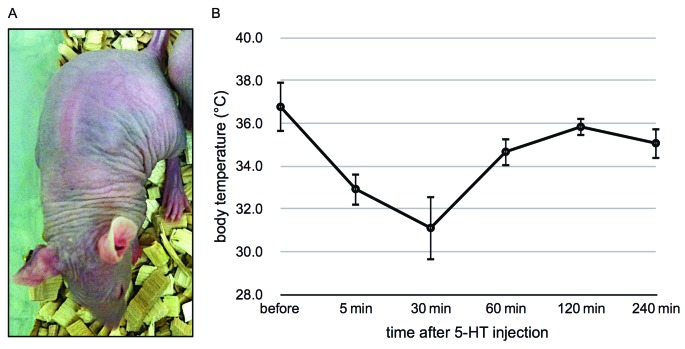
Physiological changes in 5-HT-treated mice. (A) Mouse 5 min after 5-HT injection; (B) body temperature of 5-HT-treated mice.

The 5-HT-treated mice presented with several severe side-effects, such as depressed behavior, tremors, respiratory depression and sporadic diarrhea. The skins of the mice had also turned blue (Fig. 5A). Recovery from these side-effects was observed 1 h following treatment. Abdominal measurement of body temperature documented a decrease in body temperature to 31°C within 30 min (Fig. 5B). Two hours after injection, normal body temperature was measured. This was not observed in the EGF-stimulated mice.

## Discussion

Chemotherapeutic drugs affect cells in the S or M phase. One challenge of SCCHN chemotherapy is that a small percentage of tumor cells do not proliferate (9), and thus arrest in the G0 phase of the cell cycle. Therefore, these cells are not affected by chemotherapy. This could be one reason for tumor recurrence at a later date. The recruitment of these G0 cells into the active cell cycle and thus, proliferation, may increase the efficacy of chemotherapeutic agents.

In this study, we aimed to recruit non-cycling tumor cells into the mitotic cycle in order to sensitize them to chemotherapeutic agents. The majority of tumor cells express EGFR. They all have the potential to process the EGF signal. Our results showed that EGF stimulation enhanced tumor volume, indicating that the application of extra EGF had a proliferative effect on tumor cells. This effect did not differ in the hEGF- and mEGF-injected mice, a fact which could be explained with the homologous amino acid sequences. The increased tumor volume did not reach significance, when compared to the control tumors, a fact which could be caused by the small number of mice treated, the short treatment interval and the way of application or dosaging. The majority of resting cells was located in the center of the cell nests. Further studies are required in order to examine these cells in more detail. For tumor therapy, cells that can re-enter the cell cycle are the main focus of interest, as these cells can survive chemotherapy and lead to cancer recurrence. The arrest in the cell cycle may be caused by the large distance from the blood vessels. Consequently, chemotherapeutic agents cannot reach these cells. A treatment procedure containing various cycles of EGF and cytostatic drugs may potentially prove to be more effective.

EGFR is a multifunctional receptor involved in proliferation, motility, angiogenesis and survival of tumor cells (13). Three major pathways, PI3K/Akt, Ras/Raf/MEK/MAPK and PLC/PKC, are involved in signal transduction (7). Further analysis will show which signaling cascade is activated. EGF-stimulated xenografts have a higher risk of ulceration. It is hypothesized that EGF application increases tumor cell invasiveness.

The amplitude of growth increase depends on the number of EGF receptors on the cell surface (14). Compared with A431, Detroit 562 tumors have a 3.6-fold lower EGFR-binding activity (12,14), which may result in lower growth induction compared to A431 xenografts.

Our results are consistent with the findings of Ozawa *et al*(14) and Ginsburg and Vonderhaar (15), which showed that EGF treatment stimulated the growth of SCCHN tumor xenografts, showing an increased tumor volume in the A431, NA and Ca9-22 cell line mouse xenografts after treatment with murine EGF. Factors such as gender-specific EGF host production (16), treatment interval, dosage and the way of application (osmotic pump or injection) affect the results. All EGFR ligands own the conserved EGF motive. It is characterized by 6 cysteines that form disulfide bridges with each other (17). The length of amino acid sequences between the cysteines of hEGF and mEGF is identical (CX7CX5CX10CXCX8C, X could be any amino acid). Furthermore, the recombinant EGFs have 70% sequence homology (Fig. 1E). The tumors of the 5-HT-treated mice showed a lower growth increase compared to the control tumors. Pratesi *et al*(18) described an increase in tumor growth after administration of 200 μg/day 5-HT delivered by osmotic mini-pumps in small lung cancer cell xenografts. 5-HT could have a dose-dependent effect, showing an inhibition of tumor growth when administered at lower doses (20 μg/day). One possible reason for tumor growth decrease could possibly be the reduction of blood flow in tumor vessels by 5-HT which impairs oxygen supply (19). 5-HT-treatment possibly does inhibit tumor growth via impact on tumor-feeding vessels. The decrease in tumor blood flow and, consequently, a deficit in oxygen and nutrient supply reduce the proliferation tempo (8). This effect would overcome the prospective pro-mitotic effect of 5-HT on tumor growth. 5-HT is not useful for our strategy to enhance proliferation. Our observations suggest vertical invasion of tumors. A possible active migration of tumor cells into the skin could destroy its structure. The administration of EGF may lead to an increased tumor cell motility. Seventy-four percent of the *in vitro*-cultured Detroit 562 cells were Ki67-positive (Fig. 4). In tumor xenografts, the majority of tumor cells was also Ki67-positive. The results indicate that these cells are in the active cell cycle. A number of Ki67-negative cells was localized in the middle of the tumor cell nests. These cells were supposed to be quiescent. In the EGF-stimulated mice, the number of Ki-67 positive cells was much higher. The size of cell nests correlated with the number of inner Ki-67-negative cells. It is possible that these resting cells are more differentiated. Another explanation could be that they do not have enough resources for mitosis. Oxygen diffuses into tissue and reaches a distance of approximately 100 μm. Due to the large distance to the feeding arterioles, these cells may stop cell cycling to prolong their survival (20). Most Ki67-positive cells also express high levels of EGFR. The Ki67-negative cells have reduced levels of EGFR.

In conclusion, in the present study we show that it is possible to stimulate tumor cells by EGF, and thus enhance cell proliferation, resulting in a higher tumor growth compared to the untreated control group. In our future investigations, we plan to include a higher number of mice and an adjustment of the EGF dosage, considering the heterogeneity of SCCHN tumors. Furthermore, we plan to treat EGF-stimulated SCCHN mice with chemotherapeutic drugs to investigate whether these mice show a better responce to therapy compared to a non-stimulated control group. These data may result in new clinical, stratified therapy regimes.
